# Psychometric properties of the Persian version of Proactive-Safety Role Orientation questionnaire (PRO-SAFE)

**DOI:** 10.1186/s40359-023-01474-y

**Published:** 2023-12-09

**Authors:** Reza Jafari Nodoushan, Gholam Hossein Halvani, Reyhane Sefidkar, Hamidreza Mokarami, Mahdi Jafari Nodoushan

**Affiliations:** 1 Department of Occupational Health Engineering, School of Public Health, Shahid Sadoughi University of Medical Sciences, Yazd, Iran; 2 Center for Healthcare Data Modeling, Departments of Biostatistics and Epidemiology, School of Public Health, Shahid Sadoughi University of Medical Sciences, Yazd, Iran; 3 Department of Ergonomics, School of Health, Shiraz University of Medical Sciences, Shiraz, Iran

**Keywords:** Safety, Behavior, Self-efficacy, Motivation, Psychometrics

## Abstract

**Background:**

Participation and initiative of workers are effective in promoting safety in the workplace. Proactive-Safety Role Orientation questionnaire (PRO-SAFE) is a proper tool to evaluate the psychological drivers that support the proactive orientation of individuals toward workplace safety. This study was conducted to translate and measure the psychometric properties of the Persian version of PRO-SAFE.

**Methods:**

The PRO-SAFE was translated into Persian using procedures for translation and cross-cultural adaptation. To collect data, 252 employees of a steel complex were selected. To measure the validity of the questionnaire, face, content, convergent, and construct validity was utilized. The questionnaire’s reliability was evaluated by assessing its internal consistency.

**Results:**

The mean of the content validity index and content validity ratio was equal to 0.83 and 0.85, respectively. We found a positive correlation between PRO-SAFE and safety behavior dimensions (*r* = 0.372 to 0.792, *P* < 0.001). Confirmatory factor analysis showed the Persian version of the PRO-SAFE questionnaire had an excellent six-factor model consistent with the original questionnaire. Cronbach’s alpha of the Persian version of the PRO-SAFE questionnaire was obtained between 0.717 to 0.880.

**Conclusions:**

The Persian version of the PRO-SAFE questionnaire was found to have appropriate psychometric properties, indicating that it can be confidently used as a valid tool for assessing proactive role orientation toward safety management among Iranian employees.

## Background

The occurrence of occupational accidents in workplaces is considered an important problem for countries. The occurrence of occupational accidents in workplaces can have various health, social, and economic consequences for the workplace, workers, and their families [1]. International reports show that every year 78 million workers die due to occupational accidents and 374 million workers have a non-fatal occupational accident [2]. The results of a study in Iran showed that during the years 2007–2016, 207,604 workers suffered occupational accidents. Also, the death rate due to occupational accidents during 10 years was 0.4 to 0.6% [3].

Overall, occupational incidents and their negative impacts have drawn attention to safety in workplaces. To increase the level of safety at the workplace, different methods and approaches are being considered [4, 5]. One of the most important is the proactive approach and monitoring of employee safety behavior. In various studies, a variety of psychological factors have been considered for the participation of employees in expanding and improving the safety of the workplace [6, 7]. The review of studies shows that many authors have emphasized the motivation and proactive participation of individuals in improving the level of safety [8, 9]. Attention to factors that help increase employee motivation and participation can be effective in improving the level of safety and reducing the number of incidents in the workplace [10, 11]. Safety motivation can improve safety outcomes by affecting the safety behavior of employees [12]. In the study of Liu et al., the relationship between safety behavior and occupational accidents and injuries was confirmed [13].

Given the importance attached to employee motivation in various studies, it is necessary to identify and explain the psychological and social mechanisms that affect motivation and proactive safety behaviors [11, 14]. When looking at employee safety behavior, it should not be assumed that individuals are passive about external expectations. In this regard, attention should be paid to understanding, cognition, emotions, and other psychological factors affecting employee motivation [11, 15].

Curcuruto et al. defined and validated the Proactive-Safety Role Orientation questionnaire (PRO-SAFE) to assess the psychological drivers of a proactive tendency toward safety management in the workplace. In reviewing the literature, they examined some stable motivational states that can be effective on people’s active behavior and tendencies in a specific field. After reviewing the studies, they selected six psychological drivers to further investigate and determine their relationship with safety. These six factors included role breadth self-efficacy (SE), control perception (CP), psychological ownership (PO), felt responsibility (FR), anticipation orientation (AO), and improvement orientation (IO) [11].

The concept of role breadth self-efficacy is the extent to which a person is confident in their ability to participate in a certain task. Research findings show that role breadth self-efficacy is related to outputs such as proactive work performance [11, 16]. Perceived control is defined as the belief of an individual to influence operational and organizational processes. In this regard, Curcuruto et al. defined perceived control as the degree to which people believe they can influence safety processes [11, 17]. They also define psychological ownership as people’s understanding and feeling that the issue of safety is personal and that they own the safety of the workplace. In other words, individuals understand the safety of the workplace as a personal concern and take action in case of problems in the implementation of safety programs [11]. Personal responsibility is another motivational construct that has been considered an important antecedent of behavior in various studies [17, 18]. Curcuruto et al. defined felt responsibility as a measure to assess people’s willingness to participate in safety programs beyond their official duty. They also defined anticipation orientation as a forward-looking mindset to anticipate critical situations and potential safety hazards. Forward-looking people are more interested in managing risks and preventing harm. Another psychological driver is improvement orientation. Curcuruto et al. Considered safety improvement orientation and defined it as the desire of individuals to improve safety beyond standards [11].

So far, some of these factors have been investigated and their relationship to safety indicators has been examined. In the study of Wang et al., the felt safety responsibility was investigated in Chinese construction workers and their supervisors. They reported that Cronbach’s alpha was 0.884 for the 4-item felt safety responsibility scale [15]. In another study [19], Cronbach’s alpha was 0.887 for the dimension of felt safety responsibility.

A review of international studies shows that in recent years, attention has been paid to the safety behavior of employees in industries. Also, the effect of psychological drivers on the safety behaviors of employees has been confirmed. In domestic studies, the influence of organizational factors on safety behaviors has been investigated, but the role of psychological drivers has been investigated less. PRO-SAFE can evaluate the psychological drivers toward proactive safety among employees. To the best of our knowledge, PRO-SAFE has not been formally translated into Persian. Therefore, its psychometric properties have not been investigated yet. This study aimed to develop the Persian version of the PRO-SAFE questionnaire and measure its psychometric properties among Iranian workers.

## Methods

### Design of study and population

The current study was a cross-sectional study that was conducted among the employees of Yazd Steel Complex during the winter and spring of 2023. The inclusion criterion was to have Persian as a mother tongue and have at least 1 year of job experience. Therefore, all employees with job experience of at least 1 year were invited to the study. Individuals who did not want to participate in the study were excluded. Also, some questionnaires were removed due to incompleteness or misleading answers. Two hundred eighty-three out of the 320 workers volunteered to participate in the study and completed a written consent form and questionnaire (response rate: 88%). After reviewing the questionnaires, 31 questionnaires were removed and 252 questionnaires remained as the final sample. To carry out psychometric studies, 4 to 10 participants are proposed for each item [20, 21]. Therefore, the sample size was sufficient to perform psychometry. The objectives of the study, the method of answering the questionnaire, and the ethical obligations, including the confidentiality of the questionnaires, were explained to the workers.

### PRO-SAFE questionnaire

This questionnaire was presented by Curcuruto et al. This scale aims to assess the psychological factors that support the proactive orientation of individuals’ tendency to safety management. These factors are six dimensions, including SE, CP, PO, FR, AO, and IO. Each of the dimensions comprises four individual items, resulting in a total of 24 questions encompassed within the scale. The scoring of this questionnaire is on a 5-point Likert scale (1 = strongly disagree; 5 = strongly agree).

### Translation of the questionnaire

To start the translation process, correspondence was made with the designers and permission to translate and localize the questionnaire was obtained. The utilization of the Brislin model exhibits the capability of creating dependable cross-cultural instruments. Therefore, to facilitate the translation of the PRO-SAFE questionnaire, the approach of Brislin’s model was employed [22]. The initial step involved the translation of the questionnaire into Persian by two proficient experts. Then, the research team investigated the two translated scales and checked the items for ambiguity and inconsistency. Finally, at this stage, a temporary Persian version of PRO-SAFE was prepared. In the following, an English language expert who was not aware of the original version of the questionnaire translated the provisional Persian version into English (backward translation). Finally, the final version of the questionnaire was evaluated by a group of academic and industrial experts [23, 24].

### Methods used to assess validity

#### Face validity

To assess the face validity of the Persian rendition that was devised, it was given to 14 occupational health and safety specialists and psychologists specialists (10 academic specialists and 4 industry specialists) and 10 steel employees. We requested specialists and employees that review each item for comprehension, grammar, wording, and vocabulary. They evaluated each item on a 5-point Likert scale from 1(not important at all) to 5 (very important). The impact scores higher than 1.5 were considered admissible [25].

#### Content validity

Content validity was examined through the content validity index (CVI) and content validity ratio (CVR). For this purpose, a board of 16 occupational health and safety specialists and psychologist specialists (industrial and organizational psychology) was formed. To evaluate the CVI index, specialists investigated the three criteria of simplicity, clarity, and relevance for each item. According to the instruction, a CVI of less than 0.7 is unacceptable and the item must be removed. A CVI above 0.79 is appropriate, and a CVI between 0.7 and 0.79 needs revision. To evaluate the CVR index, were asked from specialists to evaluate the necessity of each item. According to Lawshe’s table, items with a CVR above 0.49 are essential (for 16 specialists) and items with a lower 0.49 should be removed [26].

#### Construct validity

To evaluate the construct validity of the Persian version of the questionnaire, confirmatory factor analysis (CFA) with the Maximum Likelihood method was used. Goodness-of-fit of the six-factor structure of PRO-SAFE were assessed through following indices: Root mean square error of approximation (RMSEA) with admissible level of smaller than 0.08, goodness-of-fit index (GFI) with acceptance level between 0.8 and 1, χ2/degree of freedom ratio (χ2/df) with acceptance level more than 2, comparative fix index (CFI) with acceptance level greater than 0.9, and incremental fit index (IFI) with acceptance level greater than 0.90 [21, 27]. Also, standardized loading factors higher than 0.3 are considered [28].

#### Convergent validity

In the present study, Neal and Griffin’s safety behavior questionnaire was used to assess the convergent validity. This questionnaire has six items and evaluates two dimensions of safety compliance and safety participation [29]. In the study of Homayounfar et al., the content validity of the Persian version of this questionnaire was confirmed. Also, Cronbach’s alpha of safety compliance and safety participation were 0.832 and 0.866, respectively [30]. All participants completed the safety behavior questionnaire and the PRO-SAFE Persian version. Cronbach’s alpha for the entire safety behavior questionnaire was equal to 0.871. Spearman’s correlation test was used to examine the correlation between safety behavior dimensions and the dimensions of the PRO-SAFE questionnaire.

#### Reliability

To evaluate the internal consistency reliability of the questionnaire, Cronbach’s alpha and split-half methods were used. Cronbach’s alpha coefficient of 0.70 or more is acceptable [31].

### Statistical analysis

In this study, the Kolmogorov-Smirnov test was used to check the data distribution. Non-parametric tests were used for variables that did not have a normal distribution. Data analysis was done utilizing SPSS software (Armonk, NY: IBM Corp), AMOS 24 (USA, SPSS Inc.)

## Results

The mean age of the participants was equal to 37.17 ± 6.02 years. The minimum and maximum age was 22 and 53 years, respectively. The mean job experience was 12.26 ± 5.65 years. The range of job experience was between 1 and 26 years. Table 1 depicts the findings about demographic information. Also, the mean and standard deviation of safety behavior and PRO-SAFE dimensions are presented in Table 2.

**Table 1:** Frequency distribution of demographic information

Variable	Classification	Frequency	Percentage
Age (years)	≤ 29	28	11.1
	30–39	129	51.2
	40–49	89	35.3
	≥ 50	6	2.4
Job experience (years)	≤ 5	43	17.1
	6–10	50	19.8
	11–20	150	59.5
	≥ 21	9	3.6
Educational background	High school	35	13.9
	Diploma	91	36.1
	Associate Degree	33	13.1
	Bachelor	81	32.1
	MSc and above	12	4.8
Work schedule	Three-shift	192	76.2
	Day-work	60	23.8
Marital status	Single	13	5.2
	Married	239	94.8

**Table 2:** Descriptive statistics of the Persian version of PRO-SAFE and safety behavior

Variable	Dimensions	Mean (Standard deviation)	Range
Psychological drivers	Role breadth self-efficacy	3.52 (0.84)	1–5
	Perceived control	3.57 (0.78)	1–5
	Psychological ownership	3.66 (0.79)	1–5
	Felt responsibility	3.68 (0.83)	1–5
	Anticipation orientation	3.64 (0.81)	1–5
	Improvement orientation	3.57 (0.81)	1–5
Safety behavior	Safety compliance	3.73 (0.86)	1–5
	Safety participation	3.58 (0.87)	1–5

### Validity

#### Face validity

Investigating the opinions of specialists and individuals of the target group showed that all items are clear and related. Also, the impact scores of items were above 1.5 and no item was deleted.

#### Content validity

As mentioned, two indices CVR and CVI were used in the content validity check. The mean of CVR was equal to 0.85. Also, the mean of CVI was equal to 0.83. The CVI for role breadth self-efficacy, control perception, psychological ownership, felt responsibility, anticipation orientation, and improvement orientation dimensions was equal to 0.83, 0.82, 0.84, 0.85, 0.81, and 0.83, respectively. Also, The CVR for role breadth self-efficacy, control perception, psychological ownership, felt responsibility, anticipation orientation, and improvement orientation dimensions was equal to 0.86, 0.84, 0.85, 0.87, 0.83, and 0.85, respectively. Overall, the results of the indicators show that the content validity was excellent from the specialist’s point of view.

#### Construct validity

**Fig. 1:**
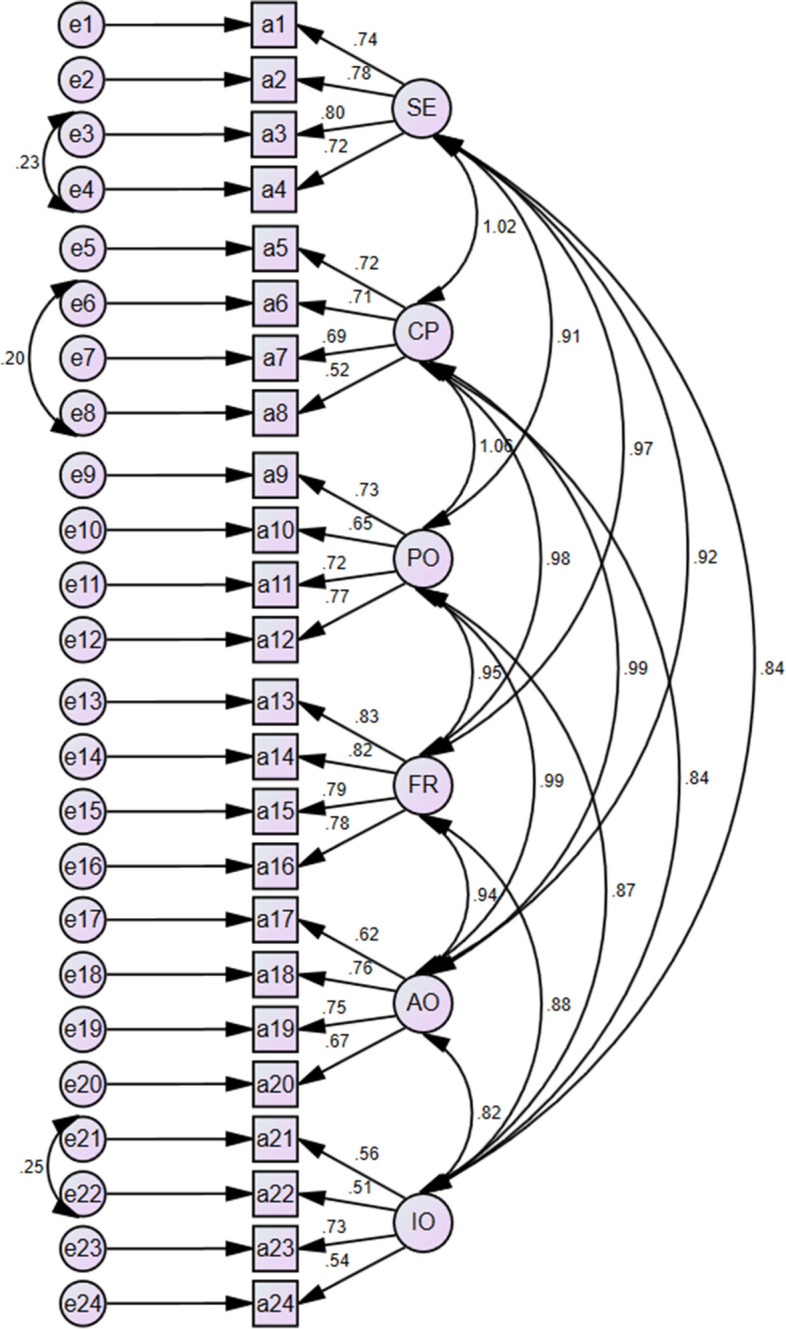
The six-factor model of the Persian version of the PRO-SAFE questionnaire obtained by Confirmatory Factor Analysis

In this study, the six-factor model of the PRO-SAFE was investigated using CFA. Path diagrams for the CFA of the six-factor model and the standardized factor loadings of the items are shown in Fig. 1. The 24 items were also well loaded into the six constructs as all the items had standardized loading factors of more than 0.3. The goodness-of-fit indices showed that the model is fitted with the data (RMSEA = 0.079, GFI = 0.840, χ2/df = 2.583, CFI = 0.902, IFI = 0.903).

#### Convergent validity

In this study, the correlation coefficient between safety behavior and PRO-SAFE dimensions was calculated. The results of this analysis are presented in Table 3. We found a positive correlation between safety behavior and PRO-SAFE questionnaire dimensions (*P* < 0.001). The lowest and highest correlation coefficients were equal to 0.372 and 0.792, respectively. The highest correlation coefficient was related to perceived control and psychological ownership dimensions. We also analyzed the inter-item correlations for items of the Persian Version of PRO-SAFE, and that inter-item correlations were acceptable (*P* < 0.001).

**Table 3:** Spearman’s correlation between safety behavior dimensions and psychological drivers

Dimensions	1	2	3	4	5	6	7	8
1- Safety compliance	1							
2- Safety participation	0.573*	1						
3- Role breadth self-efficacy	0.570*	0.588*	1					
4- Perceived control	0.566*	0.586*	0.749*	1				
5- Psychological ownership	0.573*	0.566*	0.710*	0.792*	1			
6- Felt responsibility	0.643*	0.647*	0.791*	0.699*	0.754*	1		
7- Anticipation orientation	0.581*	0.592*	0.713*	0.693*	0.737*	0.747*	1	
8- Improvement orientation	0.386*	0.372*	0.505*	0.407*	0.493*	0.572*	0.477*	1

*P < 0.001

### Reliability

The results of this study showed that the Persian version of the PRO-SAFE questionnaire has an acceptable internal consistency. Cronbach’s alpha of six dimensions and the entire questionnaire are presented in Table 4. In the split method, the items were divided into two groups. Items a1 to a12 were classified as group 1 and items a13 to a24 were classified as group 2. Guttman Split-Half Coefficient was equal to 0.932.

**Table 4:** Means, standard deviations, and reliability of the PRO-SAFE questionnaire

Dimensions	Items	Mean (SD)		Corrected item-total correlation	Cronbach’s alpha if item deleted	Cronbach’s alpha
		Item	Scale			
Role breadth self-efficacy	A1	3.49 (1.01)	14.10 (3.38)	0.641	0.836	0.854
	A2	3.56 (1.01)		0.691	0.815	
	A3	3.54 (1.04)		0.761	0.785	
	A4	3.51 (0.99)		0.688	0.817	
Control perception	A5	3.53 (1)	14.30 (3.14)	0.552	0.722	0.768
	A6	3.56 (0.94)		0.649	0.674	
	A7	3.73 (0.94)		0.570	0.714	
	A8	3.48 (1.17)		0.528	0.745	
Psychological ownership	A9	3.74 (0.94)	14.65 (10.11)	0.643	0.751	0.808
	A10	3.57 (1.06)		0.582	0.781	
	A11	3.74 (1.01)		0.608	0.767	
	A12	3.60 (0.96)		0.669	0.738	
Felt responsibility	A13	3.78 (0.97)	14.75 (3.34)	0.758	0.839	0.880
	A14	3.80 (0.93)		0.751	0.849	
	A15	3.63 (0.98)		0.757	0.839	
	A16	3.54 (1.01)		0.696	0.864	
Anticipation orientation	A17	3.61 (1.04)	14.57 (3.27)	0.529	0.765	0.786
	A19	3.55 (0.98)		0.710	0.710	
	A19	3.65 (1.01)		0.690	0.690	
	A20	3.77 (1.14)		0.767	0.767	
Improvement orientation	A21	3.52 (1.02)	14.29 (3.26)	0.567	0.617	0.717
	A22	3.50 (1.10)		0.519	0.647	
	A23	3.68 (1.01)		0.457	0.683	
	A24	3.59 (1.21)		0.483	0.672	

### PRO-SAFE development findings

In the present study, the relationship between the dimensions of the PRO-SAFE questionnaire with age and job experience was investigated using Spearman’s correlation test. The results of Spearman’s correlation test showed that there is a positive and significant relationship between age and the dimensions of role breadth self-efficacy, control perception, psychological ownership, felt responsibility, and anticipation orientation (*P* < 0.05). The strongest correlation was related to age and psychological ownership (*P* < 0.001, *r* = 0.25).

Also, The results of Spearman’s correlation test showed that there is a positive and significant relationship between job experience and the dimensions of role breadth self-efficacy, control perception, psychological ownership, and felt responsibility (*P* < 0.05). The strongest correlation was related to job experience and psychological ownership (*P* = 0.003, *r* = 0.18).

The results of the data analysis showed that there is no significant difference between the dimensions of the PRO-SAFE questionnaire and marital status, educational background, and work schedule (*P* > 0.05).

## Discussion

Presently, there has been a considerable upsurge in research regarding the focus on employee safety behaviors and safety citizenship behaviors. Psychological drivers can be effective in promoting safe behavior and active participation of individuals, so the validation of an assessment tool encourages professionals to use it in their research. During the translation process of tools, certain phrases may transform in terms of their meanings and underlying concepts owing to variations in cultures. Therefore, before implementing translated items within a new community, it is essential to ascertain their psychometric characteristics. The present investigation involved the translation and psychometric properties of the PRO-SAFE questionnaire into Persian.

The results of face validity and content validity indicators showed that the opinion of specialists and employees of the target group is satisfactory. The indicators used in CFA confirmed the model and six factors were confirmed. A suitable correlation was found between the dimensions of the PRO-SAFE questionnaire. In the present study, the dimensions of Neal and Griffin’s questionnaire (safety compliance and safety participation) had a positive correlation with the dimensions of the PRO-SAFE questionnaire. Also, the internal consistency of PRO-SAFE was confirmed.

Researchers believe that in some cases proactive safety behaviors and participation in safety programs are challenging and risky, so motivational drivers are helpful to get involved in safety programs. As mentioned, the dimensions of the PRO-SAFE questionnaire are motivational drivers that can influence proactive safety behavior. In the study of Curcuruto et al., the relationship between general PRO-SAFE measure and safety initiative, risk behaviors, safety voice, and transformational leadership was investigated. In the study of Curcuruto et al., the relationship between general PRO-SAFE measures and safety initiatives, risk behaviors, safety voice, and transformational leadership was investigated. The positive and significant relationship between the general PRO-SAFE measures with safety initiatives, safety voice, and transformational leadership was confirmed. Also, the negative and significant relationship between the general PRO-SAFE measure and risk behaviors was confirmed [11]. So far, in various studies, the relationship between some dimensions of the PRO-SAFE questionnaire and safety behavior has been investigated. In the study of Wang et al. the relationship between the felt safety responsibility dimension with safety citizenship behaviors (proactive safety behavior) was investigated and confirmed [15]. In another research, the relationship between psychological ownership and safety citizenship behavior was investigated and confirmed [32]. These results are in line with the results of the present study.

The results of the present study showed that the Persian version of the PRO-SAFE questionnaire has good and acceptable reliability. In this study, Cronbach’s alpha for the dimensions of the Persian PRO-SAFE version was between 0.717 and 0.880. The highest Cronbach’s alpha coefficient (0.880) was related to the felt responsibility dimension. In the study by Wang et al., was investigated the felt safety responsibility of construction workers in China. In their study, Cronbach’s alpha for felt safety responsibility was 0.887 which is almost equal to the results of the present study [19]. In another study, Cronbach’s alpha for felt safety responsibility was 0.884 which is almost equal to the results of the present study [15]. The study of Curcuruto et al. investigated psychological ownership in a chemical industry in Southern Europe. In their study, Cronbach’s alpha for psychological ownership was 0.88 [32]. In the present study, Cronbach’s alpha for psychological ownership was equal to 0.808.

In the CFA, the fitting indices of the model were acceptable. In the present study, RMSEA and CFI indices were obtained as 0.079 and 0.902, respectively. Curcuruto et al. investigated the overall fitting of the model in two subsamples. Their first sample was the employees of a chemical industry (plastic production) in northern Italy. Also, the second sample included workers from a manufacturing plant in northern Italy. In the chemical sample, the RMSEA and CFI indices were equal to 0.05 and 0.94, respectively. Also, In the manufacturing sample, the RMSEA and CFI indices were equal to 0.06 and 0.94, respectively. In the study of Curcuruto et al., different factor classification models (as proactive motivation and future orientation) were examined, but their results showed that the PRO-SAFE questionnaire may be used as a general measurement tool of the general concept of active role orientation towards safety management. This result showed consistency in the two investigated industrial samples and it is consistent with the results of our study [11].

It should be noted that other factors such as organizational factors, individual characteristics, and background factors may affect active behaviors by interfering with psychological drivers. PRO-SAFE provides an overview of the psychological drivers of proactive safety toward a safe workplace. Therefore, depending on the situation, some drivers may be more relevant than others, which requires further investigation.

## Limitations

The present study was conducted in a steel complex and the target population of the current research were employees of this steel complex. Also, regarding some practical reasons, the test-retest reliability was not done among respondents. Other limitations of the present study include the cross-sectional nature of the study and the possibility of bias in self-report tools. Given the social differences between distinctive countries, it is suggested that this questionnaire be translated and assessed in other nations as well.

## Conclusions

The results of the present study showed that the Persian version of the PRO-SAFE questionnaire is a reliable and valid tool for evaluating the psychological drivers of a proactive tendency toward safety management in the workplace. Therefore, this tool can be used to evaluate the psychological drivers and overall concept of proactive roles among Iranian employees.

## Data Availability

The datasets used and/or analyzed during the current study are available from the corresponding author upon reasonable request.

## Abbreviations

PRO-SAFE: Proactive-Safety Role Orientation questionnaire
SE: Role Breadth Self-efficacy
CP: Control Perception
PO: Psychological Ownership
FR: Felt Responsibility
AO: Anticipation Orientation
IO: Improvement Orientation
CVI: Content Validity Index
CVR: Content Validity Ratio
CFA: Confirmatory Factor Analysis
RMSEA: Root Mean Square Error of Approximation
GFI: Goodness-of-Fit Index
χ2/df: Chi-Square/Degree of freedom ratio
CFI: Comparative Fix Index
IFI: Incremental Fit Index
